# 2172: Association between CYP450 polymorphisms and the use of complementary medicine among patients with drug-resistant epilepsy in Puerto Rico

**DOI:** 10.1017/cts.2017.206

**Published:** 2018-05-10

**Authors:** Bianca A. Torres-Hernández, Miriam E. Ríos Motta, Adrián Llerenaes, Jorge Duconge

**Affiliations:** University of Puerto Rico-Medical Sciences Campus, San Juan, Puerto Rico

## Abstract

OBJECTIVES/SPECIFIC AIMS: Patients with epilepsy often combine their antiepileptic drugs (AEDs) with complementary medicine (CM). They use CM to treat their symptoms of comorbidities disorder, to reduce the side effect of the AEDs or trying to achieve better control of their seizures. However, the inconsistent patters of the use of CM among countries have been attributed to cultural and socio-economic factors and limited studies have explored biological factors. The aim of this study is to determinate whether or not there is an association between having genetic polymorphisms on candidate pharmacogenes for drug-metabolizing enzymes cytochrome P450 (CYP) and the use CM among patients with drug-resistant epilepsy (DRE). METHODS/STUDY POPULATION: In this cross-sectional study, patients will be recruited in the Epilepsy Clinic in the Medical Science Campus of University of Puerto Rico and in private Neurology clinics. To participate in this study, patients need to have both parents of Puerto Rican origin to be defined as Puerto Rican and have a diagnosis of DRE, defined as persistent seizures after at least 2 good trials of the proper drugs at the right dose. After the patient sign, the inform consent, a buccal swap will be collected, and the patient will complete a questionnaire. In the questionnaire, the patient will do a self-report about the use of CM (including natural products, meditation, yoga, and others), frequency of use and socio-economic information. Polymorphisms for CYP 2D6, 2C9, 2C19, or 1A2 will be determined using TaqMan^®^ SNP Genotyping Assays. Data analysis will include descriptive statistical, χ^2^ and ANOVA test. RESULTS/ANTICIPATED RESULTS: We expected to determine the frequency distribution of functional polymorphisms on CYPs among patients with DRE who are either using CM and AEDs or standard care (AEDs). Quantified the use of CM and ascertain if there is an association with the CYPs polymorphisms. DISCUSSION/SIGNIFICANCE OF IMPACT: This study is novel, because we will use an objective test, pharmacogenetics approach to rule out biological factors associated with the use of Complementary Medicine by patients’ DRE. The study will provide evidence for prospective study using specifics Complementary Medicine guiding by genotyping.

